# Supplementary material for: Spatiotemporal contact between peroxisomes and lipid droplets regulates fasting-induced lipolysis via PEX5
Source: Nat Commun. 2020 Jan 29;11:578. doi: 10.1038/s41467-019-14176-0 (PMC6989686; doi:10.1038/s41467-019-14176-0)
Supplement: Supplementary file 1 — Supplementary Information [file 41467_2019_14176_MOESM1_ESM.pdf]

Supplementary Information

Spatiotemporal contact between peroxisomes and lipid droplets regulates fasting-induced lipolysis via PEX5

Kong et al.

Supplementary Figure 1

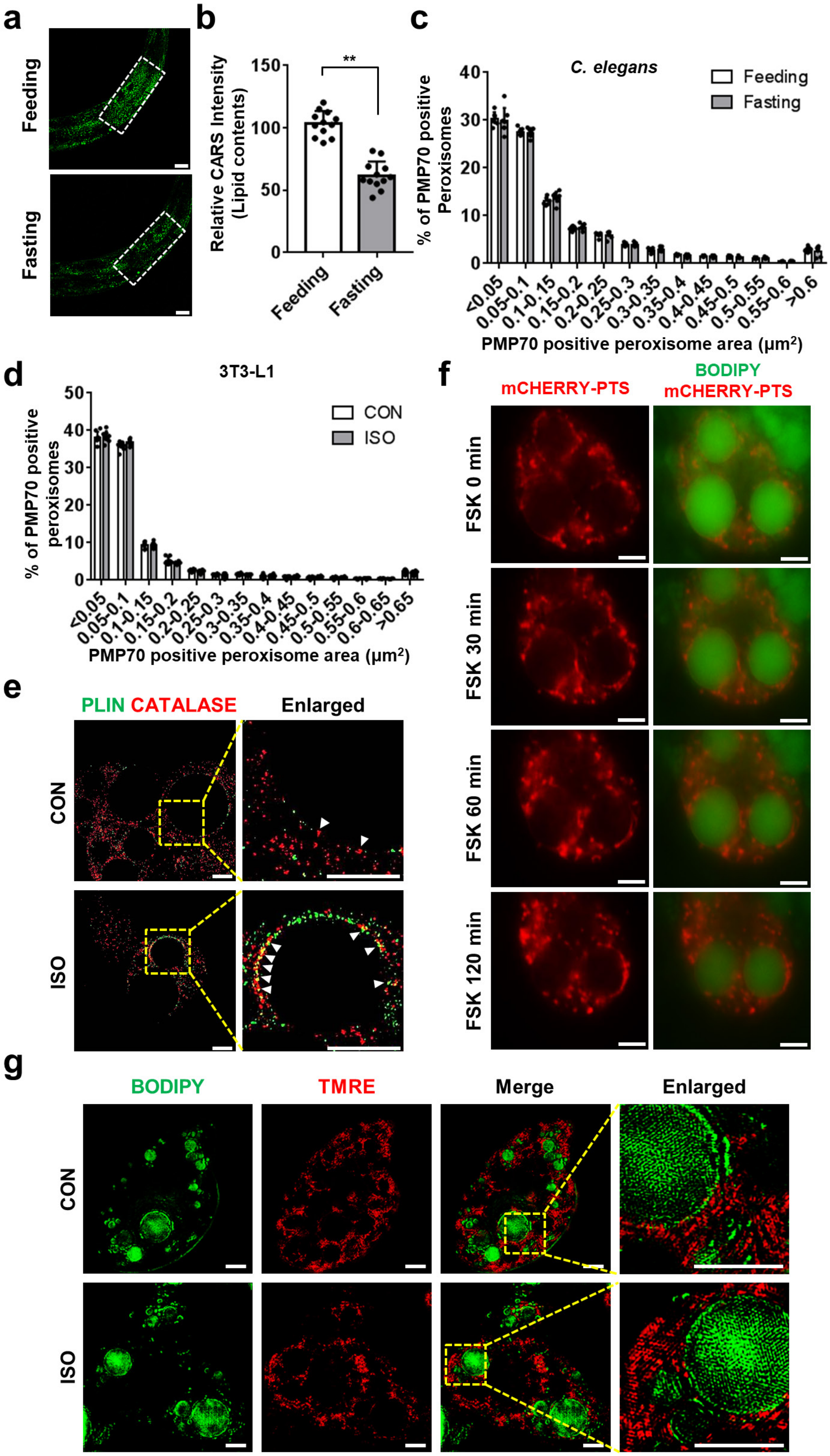

Supplementary Figure 1. Fasting increases peroxisome movement onto LDs

a, Representative CARS images in the *C. elegans* anterior intestine upon fasting (4 h). Scale bars, 50 μm. **b**, Quantification of LD contents (relative CARS intensity) calculated using Leica software (LAS X). n=12 worms. **c, d**, Quantification of PMP70 fluorescence positive peroxisomes that were classified by size using LAS X software. n=7 worms for (c), n=8 cells for (d). **e**, Representative SIM images of the localization of peroxisomal matrix protein (Catalase) and LD in differentiated 3T3-L1 adipocytes stained for endogenous Catalase (Red) and PLIN1 (green). Adipocytes were treated with or without ISO (1 μM) for 1 h. Arrowhead: contact between CATALASE and LD. Scale bars, 5 μm. CON, control; ISO, isoproterenol. **f**, Representative live images of peroxisome movement during FSK treatment (2 h) in differentiated adipocytes expressing mCHERRY::PTS1 (peroxisome marker, red) and stained with BODIPY (LD marker, green). FSK, forskolin. Scale bars, 10 μm. **g,** Representative SIM images of live adipocytes stained with BODIPY (LD marker) and TMRE (tetramethylrhodamine ethyl ester, mitochondria marker). Live cells were treated with or without ISO (1 μM) for 1 h. Scale bars, 5 μm. CON, control; ISO, isoproterenol. Data represent the mean ± SD; $^{**}P < 0.01$ (unpaired two-tailed student's *t*-test).

Supplementary Figure 2

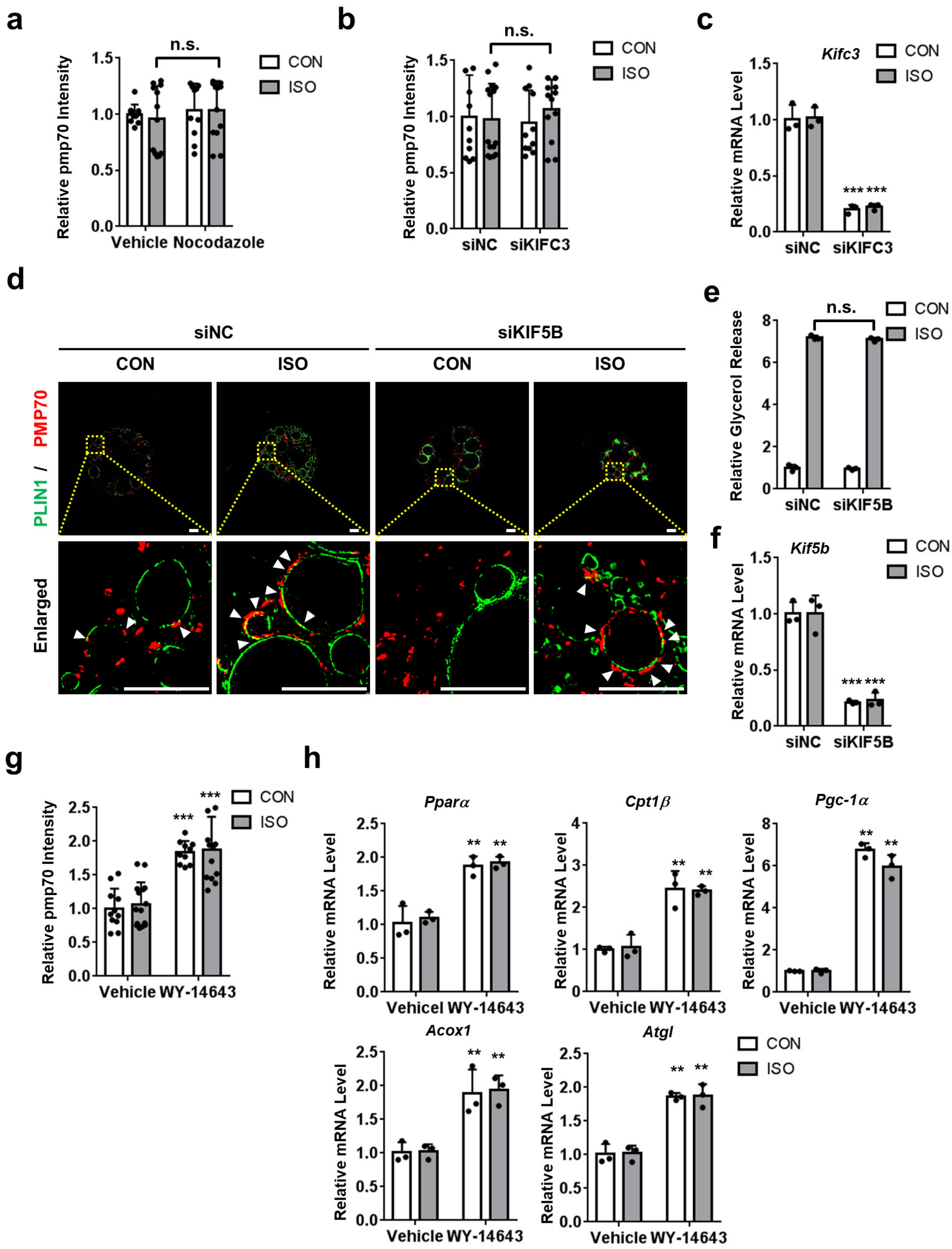

Supplementary Figure 2. Peroxisome movement is required for fasting-induced lipolysis

a, Quantification of PMP70 intensity in differentiated adipocytes. Cells were treated with or without nocodazole (0.05 μg/ml). n=10 cells for CON group; n=12 cells for ISO group; n=10 cells treated with nocodazole; n=12 cells treated with nocodazole and ISO. **b**, Quantification of PMP70 intensity in differentiated adipocytes transfected with or without siNC or siKIFC3. n=11 cells for siNC group; n=15 cells for siNC treated with ISO; n=11 cells for siKIFC3 group; n=12 cells for siKIFC3 treated with ISO. **c**, Relative *kifc3* mRNA levels in adipocytes after siRNA transfection (48 h). n=3 for each group. **d**, Representative SIM images of peroxisome-LD contacts (arrowhead) stained with PLIN1 (green) and PMP70 (red). Adipocytes were transfected with or without siNC or siKIF5B for 48 h. Scale bars, 5 μm. **e**, Concentration of released glycerol from adipocytes transfected with or without siNC or siKIF5B for 48 h. n=3 for each group. **f**, Relative *Kif5b* mRNA levels in adipocytes 48 h after siRNA transfection. n=3 for each group. **g**, Quantification of PMP70 intensity in differentiated adipocytes. n=11 cells for vehicle group; n=15 cells for vehicle treated with ISO; n=10 cells treated with WY-14643; n=13 cells treated with WY-14643 and ISO. Adipocytes were treated with or without WY-14643. **h**, Relative mRNA levels of *Cpt1β*, *Pgc-1α*, *Acox1* (PPARα target genes), and *Atgl gene* in cells treated with or without WY-14643. n=3 for each group. CON, control; ISO, isoproterenol. Cells were treated with ISO (1 μM) for 1 h. Data represent the mean ± SD; $^{***}P < 0.001$ vs. siNC-CON and $^{\#\#\#}P < 0.001$ in two-way ANOVA followed by Turkey's post-hoc test. n.s., not statistically significant.

Supplementary Figure 3

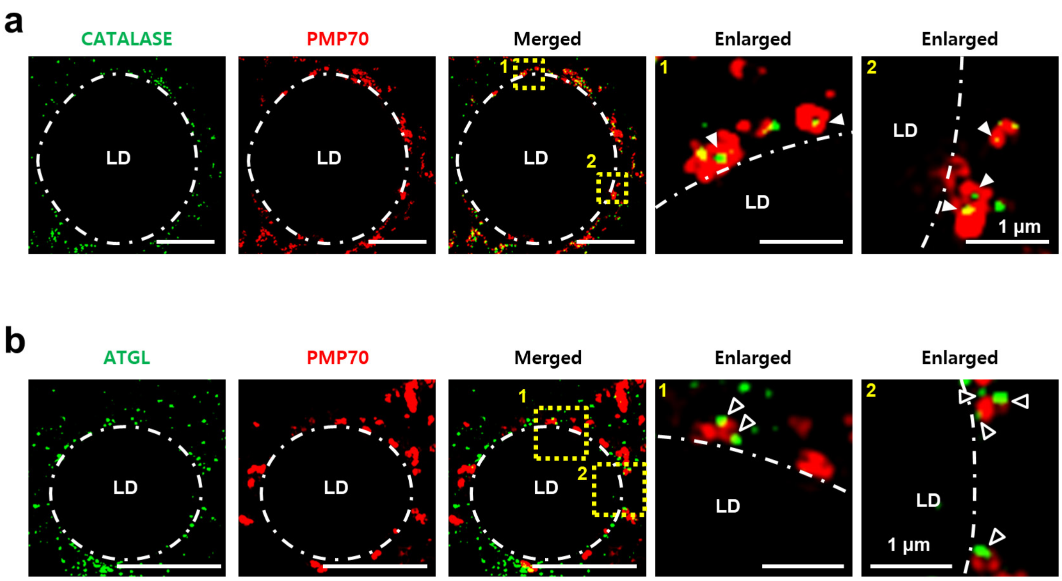

Supplementary Figure 3. ATGL and peroxisomal matrix protein localize differently in the peroxisome during fasting

a, Representative SIM images of peroxisomal matrix protein (Catalase) at LD surface in differentiated 3T3-L1 adipocytes stained for endogenous Catalase (green) and PMP70 (red) and treated with ISO. Closed arrowhead: Catalase localization in the peroxisome matrix. **b**, Representative SIM images of ATGL at LD surface in differentiated 3T3-L1 adipocytes stained for ATGL (green) and PMP70 (red) and treated with ISO. Open arrowhead: ATGL localization at the surface of peroxisome. CON, control; ISO, isoproterenol. cells were treated with ISO (1 μM) for 1 h. Scale bars, 5 μm except for enlarged images.

Supplementary Figure 4

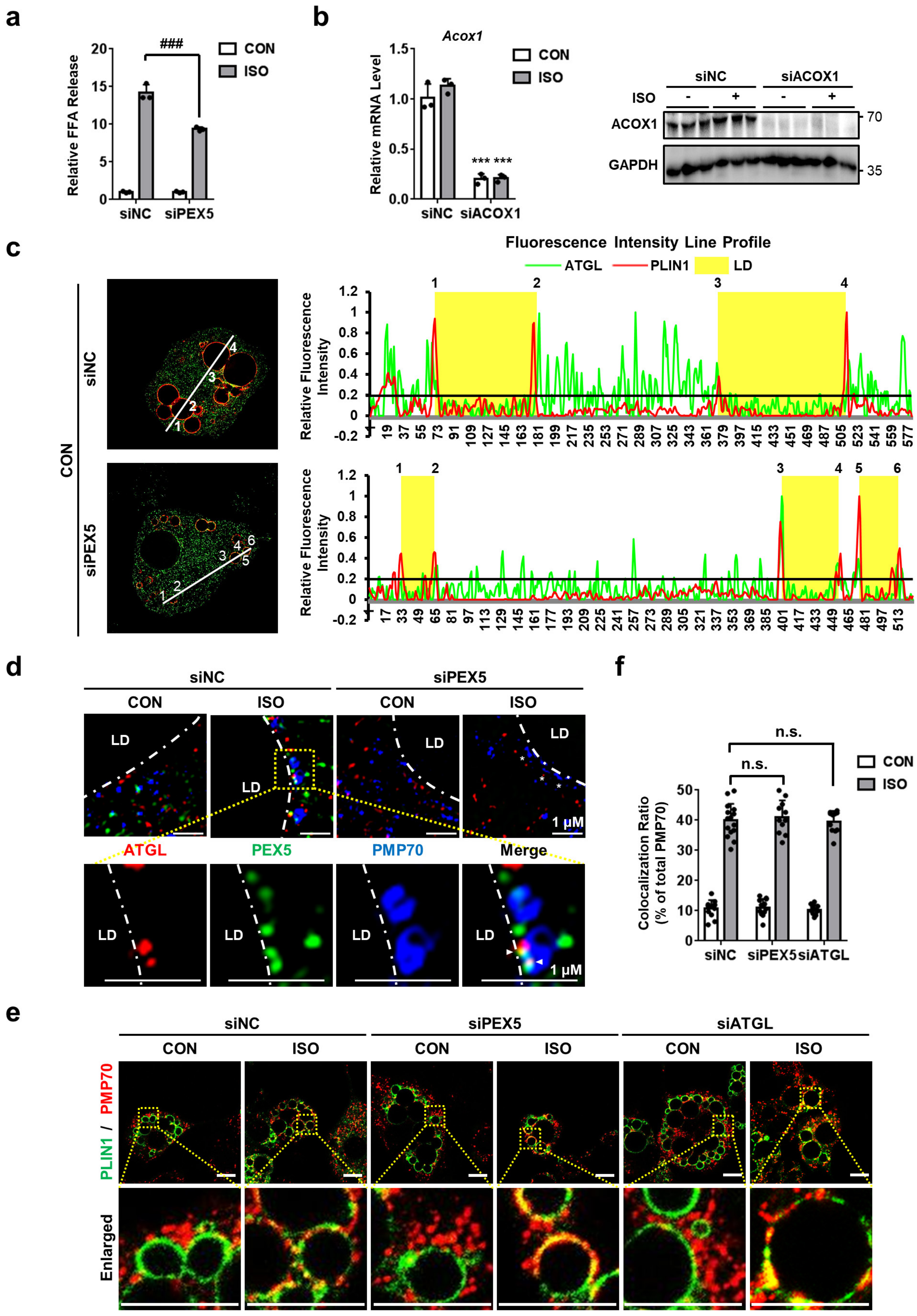

Supplementary Figure 4. PEX5 is required for ATGL translocation onto LD upon fasting
a, Concentration of released FFA from adipocytes transfected with siNC or siPEX5 for 48 hours. n=3 for each group. **b**, Relative mRNA levels of *Acox1* (left) and protein levels of ACOX1 (right) in adipocytes 48 h after siRNA transfection. **c**, Representative SIM z-section images (left) and fluorescence intensity profile from the indicated line scan (right) in Fig. 5F. Below 0.2 fluorescence intensity indicates background fluorescence signal. The LD area is highlighted in yellow box. **d,** Representative SIM images of ATGL, PEX5, peroxisomes and LDs in differentiated 3T3-L1 adipocytes stained for endogenous PEX5 (green), ATGL (red), and PMP70 (blue). Surface of LDs were indicated from DIC images. Arrowhead: Colocalization between PEX5 and ATGL at the contact points between peroxisomes and LDs. Scale bars, 1 μm. **e,f**, Representative confocal images and quantification of peroxisome-LD contacts (arrowhead) immunostained with PLIN1 (green) and PMP70 (red) in differentiated adipocytes. Cells were transfected with siNC, siPEX5, and siATGL for 48 h. n=12 cells for siNC group; n=15 cells treated with ISO; n=12 cells for siPEX5 group; n=11 cells for siPEX5 treated with ISO; n=10 cells for siATGL group; n=11 cells for siATGL treated with ISO. scale bars, 10 μm. CON, control; ISO, isoproterenol. Cells were treated with ISO (1 μM) for 1 h. Data represent the mean ± SD; $^{\#\#\#}P < 0.001$, $^{***}P < 0.001$ vs. siNC-CON in two-way ANOVA followed by Turkey's post-hoc test. n.s., not statistically significant.

Supplementary Figure 5

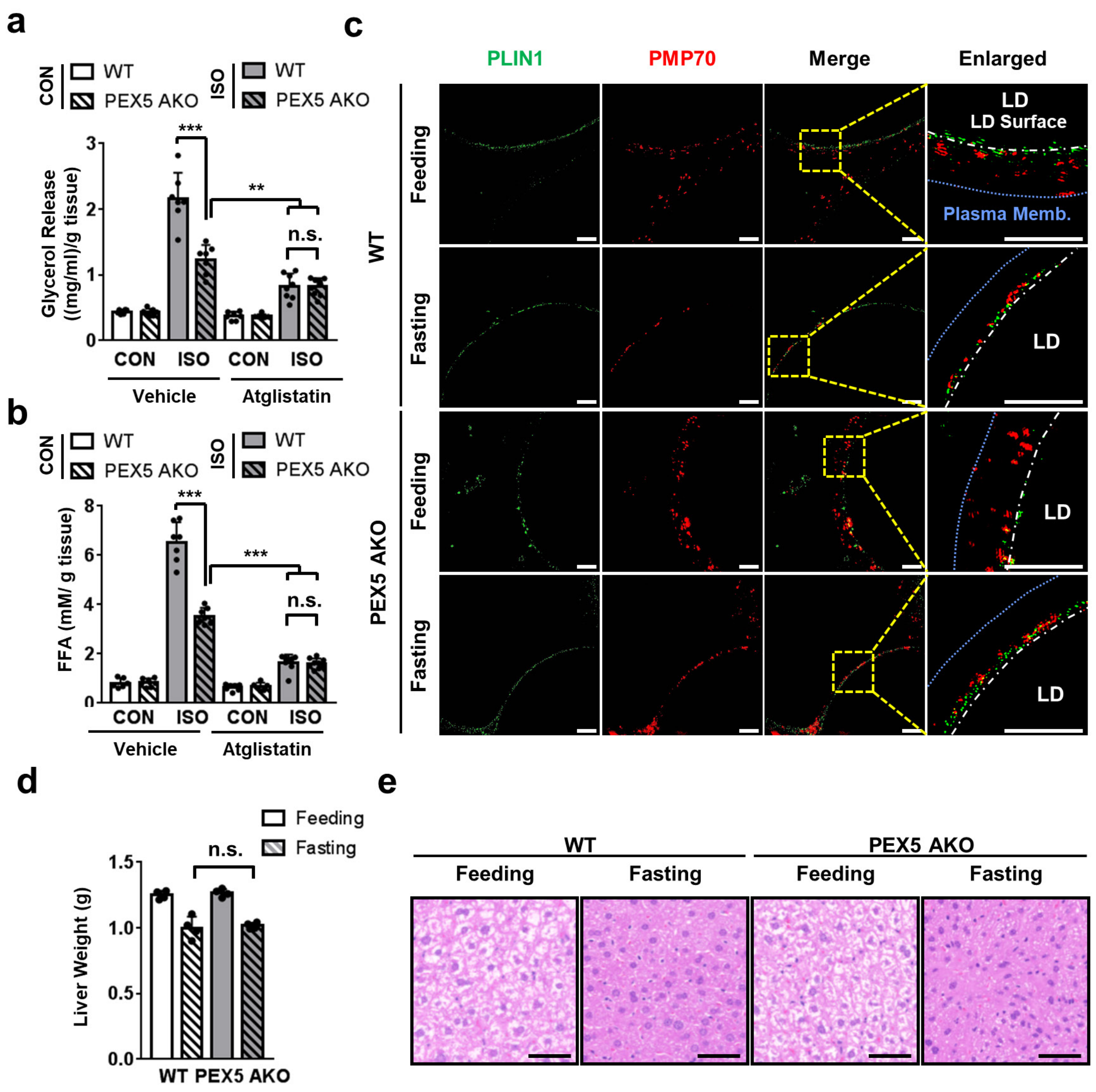

Supplementary Figure 5. Fasting-induced lipolysis is impaired in PEX5 AKO mice

a,b, *Ex vivo* lipolysis measured by glycerol (a) and FFA (b) release from eWAT treated with or without Atglistatin (50 μM) or ISO (5 μM) treatment. n=6 for CON from WT; n=7 for CON from PEX5 AKO; n=7 for WT treated with ISO; n=7 for PEX5 AKO treated with ISO; n=7 for WT treated with Atglistatin; n=6 for PEX5 AKO treated with Atglistatin; n=8 for WT treated with Atglistatin and ISO; n=9 for PEX5 AKO treated with Atglistatin and ISO. CON, control; ISO, isoproterenol. **c**, SIM images of localization of peroxisomes and LDs stained with PLIN1 (green) and PMP70 (red) from WT and PEX5 AKO eWAT under feeding and 12 h of fasting. Scale bars, 10 μm. **d**, Liver weight of WT and PEX5 AKO mice under feeding and 12 h of fasting. **e**, Representative H&E stained images of liver from WT and PEX5 AKO mice under feeding and 12 h of fasting. Scale bars, 100 μm. Data represent the mean ± SD; ****P* < 0.0001 in two-way ANOVA followed by Turkey's post-hoc test. n.s., not statistically significant.

Supplementary Figure 6

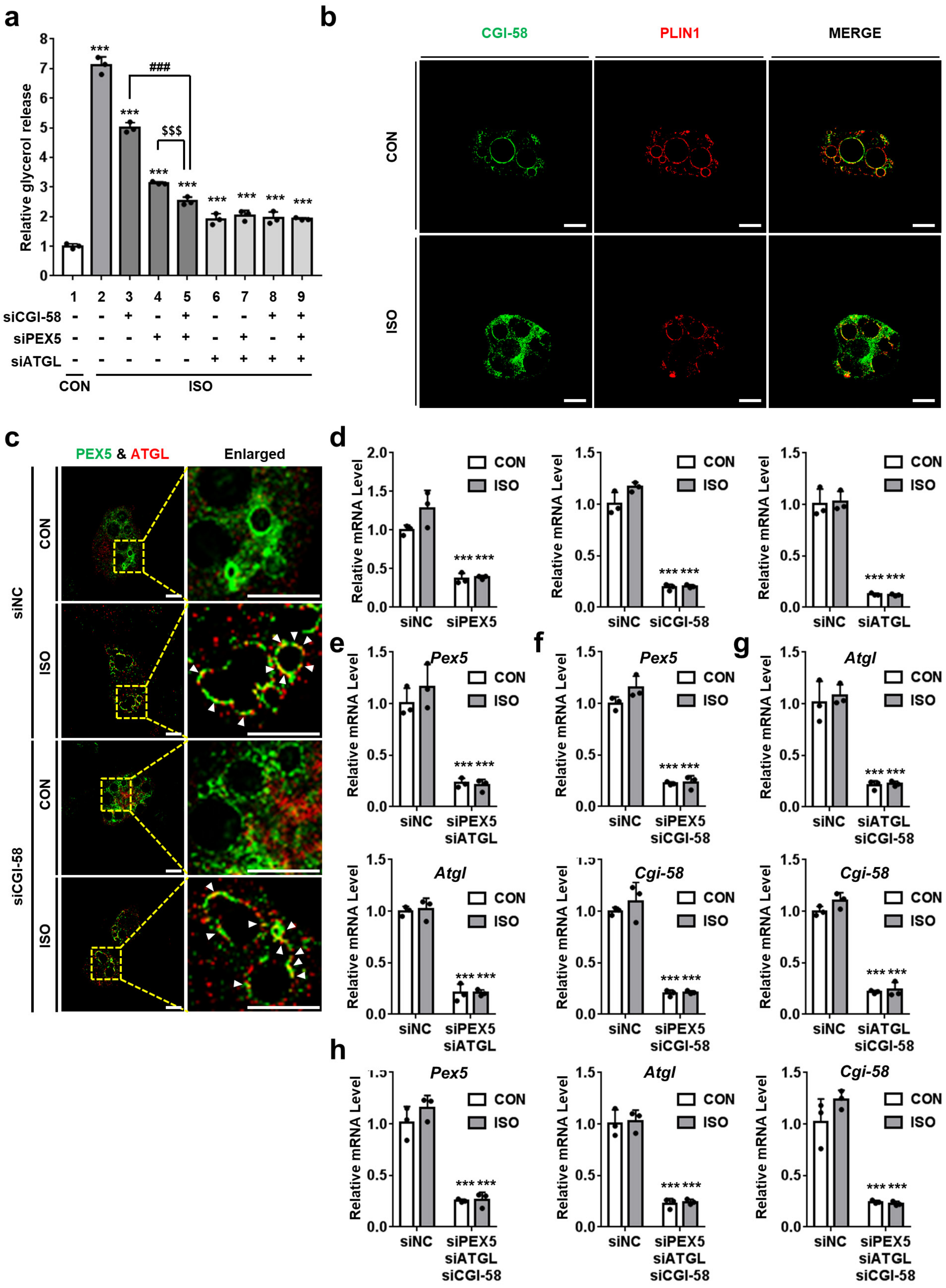

Supplementary Figure 6. PEX5 regulates fasting-induced lipolysis in a CGI-58 independent manner

a, Relative glycerol release from differentiated adipocytes after siRNA transfection (48 h). n=3 for each group. $^{***}P < 0.001$ vs. control, $^{\#\#\#}P < 0.001$ vs. siRNA transfection, $^{\$\$\$}P < 0.001$ vs. CGI-58 siRNA transfection. **b,** Representative images of adipocytes immunostained for endogenous CGI-58 (green) and PLIN1 (red). **c,** Representative SIM images of adipocytes immunostained for endogenous PEX5 (green) and ATGL (red) after NC or CGI-58 siRNA transfection (48 h). Cells were treated with ISO (1 μM) for 1 h. All scale bars, 10 μm. **d-h,** Relative mRNA levels of *Pex5*, *Cgi-58* and *Atgl* genes in adipocytes 48 h after siRNA transfection. Single siRNA transfection (d), *PEX5* and *ATGL* double siRNA transfection (e), *PEX5* and *CGI-58* double siRNA transfection (f), *ATGL* and *CGI-58* double siRNA transfection (g), *PEX5*, *ATGL* and *CGI-58* triple siRNA transfection (h). CON, control; ISO, isoproterenol. Data represent the mean ± SD; $^{***}P < 0.001$ vs. siNC-CON in two-way ANOVA followed by Turkey's post-hoc test.

Supplementary Figure 7

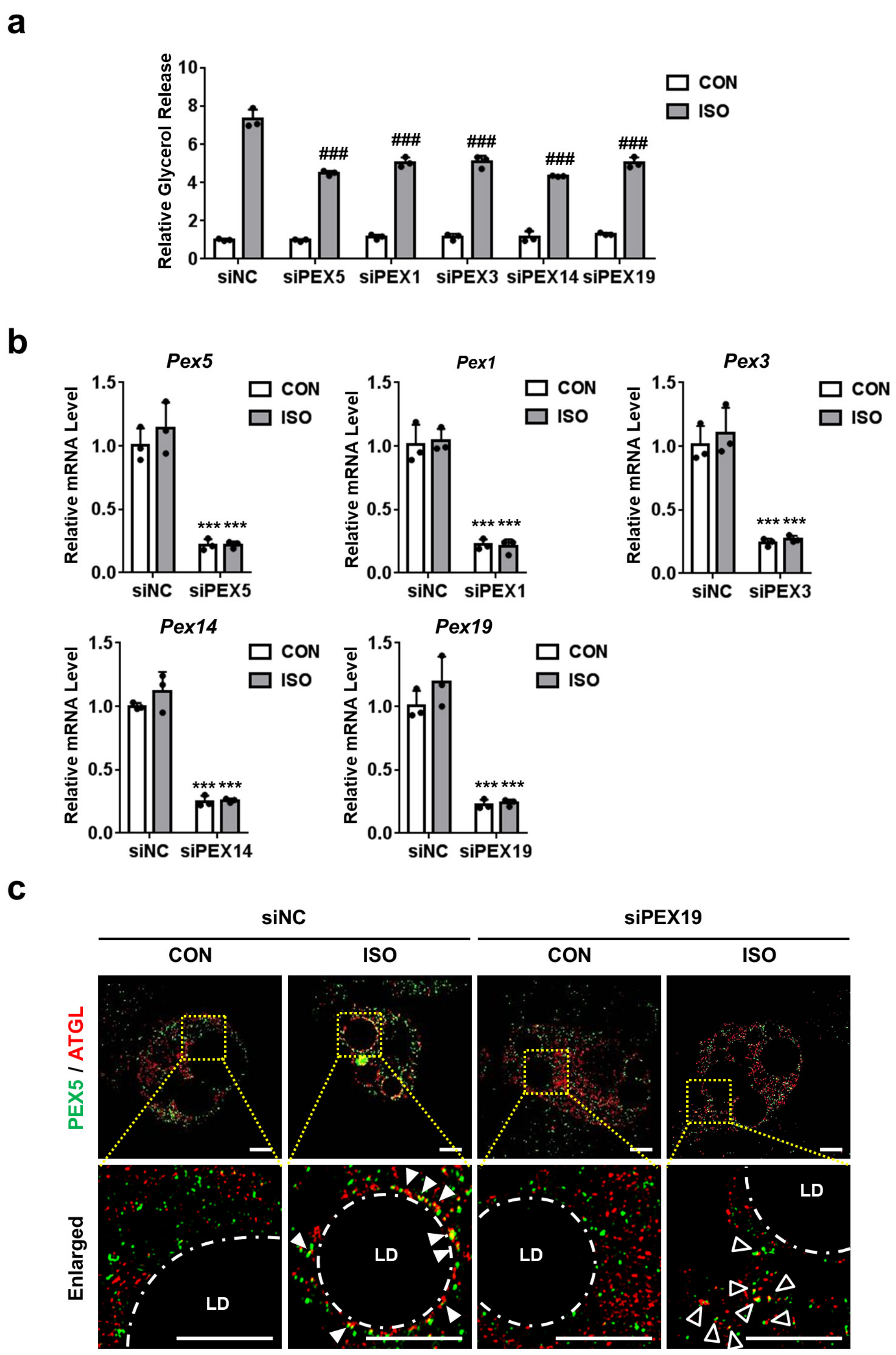

Supplementary Figure 7. peroxisomal genes would be involved in fasting-induced lipolysis in *C. elegans* and mammalian adipocytes

a, Concentration of released glycerol from adipocytes transfected with or without siNC, siPEX1, siPEX3, siPEX5, siPEX14, and siPEX19 for 48 h. n=3 for each group. Data represent the mean ± SD; $^{###}P < 0.001$ vs. siNC-ISO in two-way ANOVA followed by Turkey's post-hoc test. n.s., not statistically significant. **b**, Relative mRNA levels of *Pex1, Pex3*, *Pex5, Pex14, and Pex19 genes* in adipocytes after siRNA transfection (48 h). Data represent the mean ± SD; $^{***}P < 0.001$ vs. siNC-CON in two-way ANOVA followed by Turkey's post-hoc test. **c**, Representative SIM images of the localization of ATGL and PEX5 in differentiated 3T3-L1 adipocytes stained for endogenous PEX5 (green) and ATGL (red). Closed Arrowhead: Colocalization between PEX5 and ATGL at LD surfaces. Open arrowhead: Colocalization between PEX5 and ATGL in cytosol. Scale bars, 5 μm. CON, control; ISO, isoproterenol. cells were treated with ISO (1 μM) for 1 h.

Supplementary Figure 8. An uncropped version of the blot

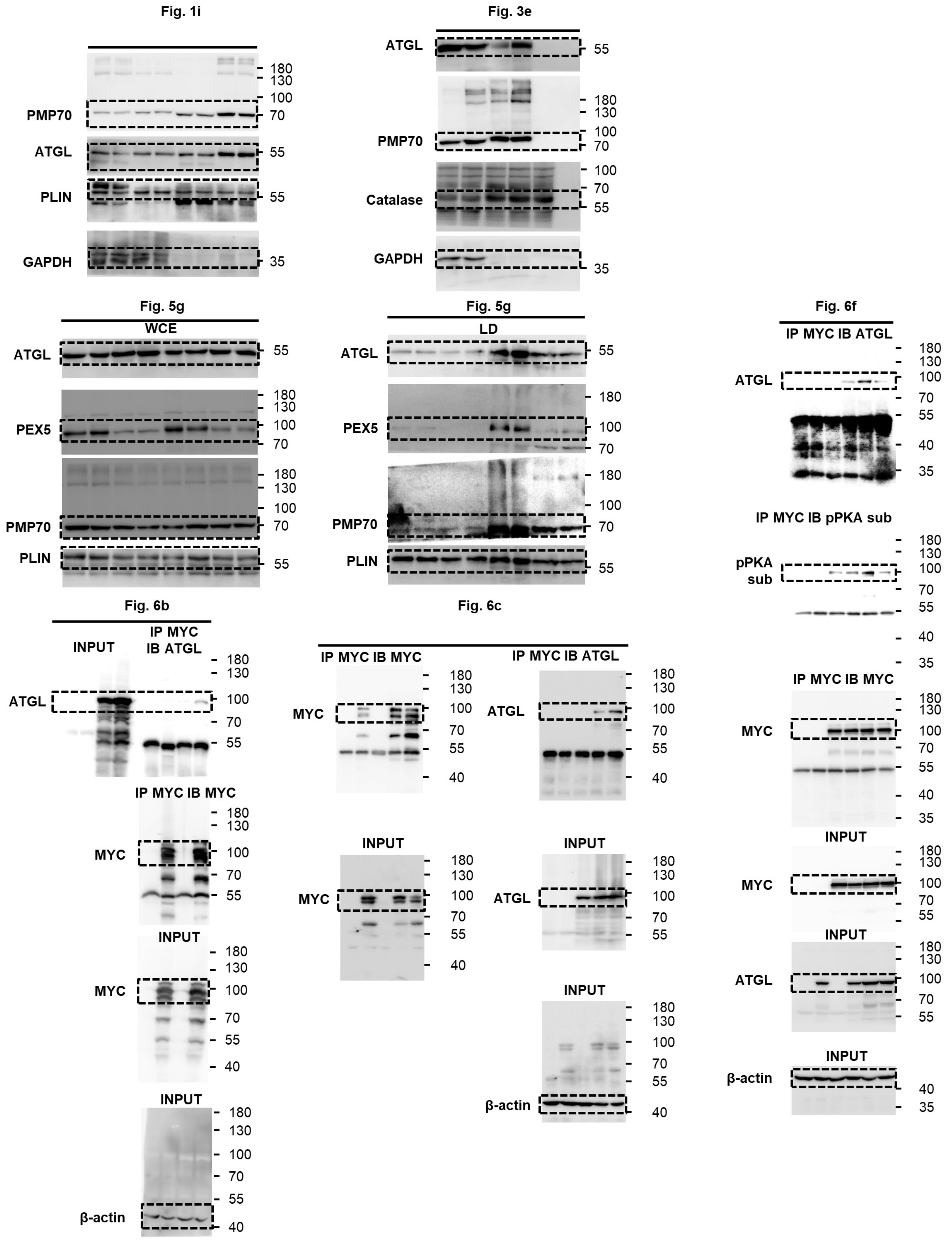

Supplementary Table 1. Sequence of primers for q-RT-PCR

Primer	Sequences
mPparα	Sense 5'- ATGCCAGTACTGCCGTTTTC<br>Antisense 5'- GGCCTTGACCTTGTTCATGT
mCpt1β	Sense 5'- TGTCATGGCAACAGTTGGTT<br>Antisense 5'- GACTCCGGTGGAGAAGATGA
mPgc-1α	Sense 5'- AAACTTGCTAGCGGTCCTCA<br>Antisense 5'- AACAATGGCAGGGTTTGTTC
mAcox1	Sense 5'- CAGGAAGAGCAAGGAAGTGG<br>Antisense 5'- CCTTTCTGGCTGATCCCATA
mAtgl	Sense 5'- TCCGAGAGATGTGCAAACAG<br>Antisense 5'- CTCCAGCGGCAGAGTATAGG
mCgi-58	Sense 5'- TGACAGTGATGCGGAAGAAG<br>Antisense 5'- AGATCTGGTCGCTCAGGAAA
mPex1	Sense 5'- TCTGCGAAATGTCAACTTGC<br>Antisense 5'- CTGTCCCTGGAGGACCATAA
mPex3	Sense 5'- ACGTGTATGCTGGTGGTTC<br>Antisense 5'- CATGCTGTAGGTCCTGCTCA
mPex5	Sense 5'- AGAACAGTGGGCAGCAGAGT<br>Antisense 5'- TGTCCCAGAAATCGACATCA
mPex14	Sense 5'- AGTCTTCATCACCCTCCAGC<br>Antisense 5'- ATCCTCCTTGTCCTCCCTCT
mPex19	Sense 5'- AGCATCATGCAGAACCTCCT<br>Antisense 5'- TGCTGCTGCTGGTACTTCTC
mKifc3	Sense 5'- GCGCAAGAAATGCCATAACGAGC<br>Antisense 5'- AGGTCACAGCATTGGTTGCCTC
mKif5b	Sense 5'- GAGTGCTGAGATTGATTCTGATG<br>Antisense 5'- CGGAGATCTGCATTATCACG

(m: mouse)
