# Supplementary material for: Spatiotemporal contact between peroxisomes and lipid droplets regulates fasting-induced lipolysis via PEX5
Source: Nat Commun. 2020 Jan 29;11:578. doi: 10.1038/s41467-019-14176-0 (PMC6989686; doi:10.1038/s41467-019-14176-0)
Supplement: Supplementary file 3 — Description of Additional Supplementary Files [file 41467_2019_14176_MOESM3_ESM.pdf]

Description of Additional Supplementary Files

File Name: Supplementary Movie 1
Description: **Peroxisomes move toward LDs upon treatment with FSK.** Merged images for live cell microscopy of peroxisome movement toward LD after FSK treatment (2 h), related to Supplementary Fig. 1f. Differentiated 3T3-L1 adipocytes were expressed with mCHERRY::PTS1 (peroxisome marker, red) and stained with BODIPY (LD marker, green). FSK, forskolin. Scale bars, 40 µm.

File Name: Supplementary Movie 2
Description: **Peroxisomes move toward LDs upon treatment with FSK peroxisome.** Peroxisome images for live cell microscopy of peroxisome movement toward LD after FSK treatment (2 h), related to Supplementary Fig. 1f. Differentiated 3T3-L1 adipocytes were expressed with mCHERRY::PTS1 (peroxisome marker, red). FSK, forskolin. Scale bars, 40 µm.

File Name: Supplementary Movie 3
Description: **Peroxisomes move toward LDs upon treatment with FSK LD.** LD images for live cell microscopy of peroxisome movement toward LD after FSK treatment (2 h), related to Supplementary Fig. 1f. Differentiated 3T3-L1 adipocytes were stained with BODIPY (LD marker, green). FSK, forskolin. Scale bars, 40 µm.
