# Supplementary material for: Spatiotemporal contact between peroxisomes and lipid droplets regulates fasting-induced lipolysis via PEX5
Source: Nat Commun. 2020 Jan 29;11:578. doi: 10.1038/s41467-019-14176-0 (PMC6989686; doi:10.1038/s41467-019-14176-0)
Supplement: Supplementary file 7 — Reporting Summary [file 41467_2019_14176_MOESM7_ESM.pdf]

nature research

Corresponding author(s): Jae Bum Kim

Last updated by author(s): Dec 3, 2019

Reporting Summary

Nature Research wishes to improve the reproducibility of the work that we publish. This form provides structure for consistency and transparency in reporting. For further information on Nature Research policies, see Authors & Referees and the Editorial Policy Checklist.

Statistics

For all statistical analyses, confirm that the following items are present in the figure legend, table legend, main text, or Methods section.

n/a	Confirmed	
☐	☒	The exact sample size (n) for each experimental group/condition, given as a discrete number and unit of measurement
☐	☒	A statement on whether measurements were taken from distinct samples or whether the same sample was measured repeatedly
☐	☒	The statistical test(s) used AND whether they are one- or two-sided<br>*Only common tests should be described solely by name; describe more complex techniques in the Methods section.*
☒	☐	A description of all covariates tested
☐	☒	A description of any assumptions or corrections, such as tests of normality and adjustment for multiple comparisons
☐	☒	A full description of the statistical parameters including central tendency (e.g. means) or other basic estimates (e.g. regression coefficient) AND variation (e.g. standard deviation) or associated estimates of uncertainty (e.g. confidence intervals)
☐	☒	For null hypothesis testing, the test statistic (e.g. F, t, r) with confidence intervals, effect sizes, degrees of freedom and P value noted<br>*Give P values as exact values whenever suitable.*
☒	☐	For Bayesian analysis, information on the choice of priors and Markov chain Monte Carlo settings
☒	☐	For hierarchical and complex designs, identification of the appropriate level for tests and full reporting of outcomes
☐	☒	Estimates of effect sizes (e.g. Cohen's d, Pearson's r), indicating how they were calculated

Our web collection on statistics for biologists contains articles on many of the points above.

Software and code

Policy information about availability of computer code

Data collection	Zen2009 (Confocal microscope, Zeiss); AcquireSR (OMX SIM microscope, GE Healthcare Life Sciences; LAS X(CARS microscope, Leica)
Data analysis	ImageJ; GraphPad Prism 7; Zen2009; SoftWoRx (OMX SIM microscope, GE Healthcare Life Sciences); LAS X

For manuscripts utilizing custom algorithms or software that are central to the research but not yet described in published literature, software must be made available to editors/reviewers. We strongly encourage code deposition in a community repository (e.g. GitHub). See the Nature Research guidelines for submitting code & software for further information.

Data

Policy information about availability of data

All manuscripts must include a data availability statement. This statement should provide the following information, where applicable:

- Accession codes, unique identifiers, or web links for publicly available datasets
- A list of figures that have associated raw data
- A description of any restrictions on data availability

The authors declare that the data supporting the findings of this study are available within the research article and its supplementary Information files. Further information is available from the corresponding author upon request.

Field-specific reporting

Please select the one below that is the best fit for your research. If you are not sure, read the appropriate sections before making your selection.

☒ Life sciences ☐ Behavioural & social sciences ☐ Ecological, evolutionary & environmental sciences

October 2018

Life sciences study design

All studies must disclose on these points even when the disclosure is negative.

Sample size	Sample size for in vivo, ex vivo, and in vitro experiments was chosen based on commonly adopted standards in the field, resulting in statistically meaningful comparisons.
Data exclusions	Experiments failed for technical reasons were excluded from data analysis.
Replication	All experiments were carried out under standard and clearly defined conditions. And all attempts at replication were successful by at least one researcher.
Randomization	Randomization was used for analyzing image data. Mice and worms from experimental and control groups were randomly selected.
Blinding	Blinding test was used for imaging experiments. And the analysis was performed in a blinded fashion whenever possible.

Reporting for specific materials, systems and methods

We require information from authors about some types of materials, experimental systems and methods used in many studies. Here, indicate whether each material, system or method listed is relevant to your study. If you are not sure if a list item applies to your research, read the appropriate section before selecting a response.

Materials & experimental systems

n/a	Involved in the study	
☐	☒	Antibodies
☐	☒	Eukaryotic cell lines
☒	☐	Palaeontology
☐	☒	Animals and other organisms
☐	☒	Human research participants
☒	☐	Clinical data

Methods

n/a	Involved in the study	
☒	☐	ChIP-seq
☒	☐	Flow cytometry
☒	☐	MRI-based neuroimaging

Antibodies

Antibodies used	Rabbit anti-ATGL, Cell Signaling, 2138S; Rabbit anti-HSL, Cell Signaling, 4107S; pHSL, Cell Signaling, 4139; Rabbit anti-pPKA substrate, Cell Signaling, 9624S; anti-PLIN1, Greenberg et al., 1991; Guinea Pig anti-PLIN1, Fitzgerald Industries, 20R-pp004; Rabbit anti-PMP70, Abcam, ab3421; Mouse anti-PMP70, Sigma, SAB4200181; Mouse anti-PEX5, Abcam, Ab125689; Rabbit anti-PEX5, Novus biological, NBP2-38443; goat anti-PEX5, Santacruz, sc-23188; Mouse anti-Catalase, Abcam, ab16771); Mouse anti-ABHD5, Novus Biologicals, H00051099-M01; Rabbit anti-GAPDH, AbFrontier, LF-PA0018; Mouse anti-GFP, Santa Cruz Biotechnology, Sc9996; Mouse anti-MYC, Cell Signaling, 9B11; Mouse anti-βactin, Sigma Aldrich, A5316.
Validation	All the antibodies were validated by manufacturer and are widely used for similar experiments by other researchers worldwide.

Eukaryotic cell lines

Policy information about cell lines

Cell line source(s)	3T3-L1 cells, ATCC; HEK 293 cells, Laboratory of Norbert Perrimon; Cos-7 cells, MSKCC stem cell core facility.
Authentication	3T3-L1 cells were authenticated by ATCC. HEK 293 cells and Cos-7 cells were obtained as pre-authenticated lines from Laboratory of Norbert Perrimon and MSKCC stem cell core facility, respectively.
Mycoplasma contamination	We used the cell lines after testing mycoplasma contamination (ROCHE 10799050001).
Commonly misidentified lines (See ICLAC register)	None.

Animals and other organisms

Policy information about studies involving animals; ARRIVE guidelines recommended for reporting animal research

Laboratory animals	In C57BL/6 background, PEX5 AKO mice were generated by crossing adiponectin Cre mice with PEX5-loxP mice (provided by Dr. Baes, KU Leuven, Leuven, Belgium). C57BL/6 male mice were purchased from SAMTACO (Seoul, South Korea) and were housed in colony cages.

	C. elegans of all strains were grown at 20°C on nematode growth medium plates seeded with E. coli OP50. N2, Bristol was used as the wild-type strain. hjIs67[atgl-1p::atgl-1::gfp] was provided by the Caenorhabditis Genetics Center (CGC).
Wild animals	The study did not involve wild animals.
Field-collected samples	The study did not involve samples collected from the field.
Ethics oversight	All experiments with mice were approved by the Seoul National University Institutional Animal Care and Use Committee (SNUIACUC).

Note that full information on the approval of the study protocol must also be provided in the manuscript.

Human research participants

Policy information about studies involving human research participants

Population characteristics	The study analyzed the Genotype-Tissue Expression (GTEx) data from GTEx consortium (Consortium, 2013). All information about characteristics of human research participants is described in Consortium, Nat Genet 45, 580-585 (2013) and website https://gtexportal.org/home/.
Recruitment	Information is described in Consortium, Nat Genet 45, 580-585 (2013) and website https://gtexportal.org/home/.
Ethics oversight	Information is described in Consortium, Nat Genet 45, 580-585 (2013) and website https://gtexportal.org/home/.

Note that full information on the approval of the study protocol must also be provided in the manuscript.
